# Nomogram integrating MRI radiomics and white matter hyperintensity grading for predicting overall survival in patients with non‐small cell lung cancer and brain metastases receiving whole‐brain radiotherapy

**DOI:** 10.1002/pro6.70077

**Published:** 2026-06-17

**Authors:** Jianan Ni, Yuefeng Lu, Haoli Xu, Xuni Xu, Mengjing Zhao, Yingnan Xue, Gang Li, Yuxia Duan

**Affiliations:** ^1^ Department of Radiology The First Affiliated Hospital of Wenzhou Medical University Wenzhou China; ^2^ Department of Radiology Shaoxing Central Hospital Shaoxing China; ^3^ Department of Chemoradiation Oncology The First Affiliated Hospital of Wenzhou Medical University Wenzhou China

**Keywords:** brain metastases, non‐small cell lung cancer, radiomics, white matter hyperintensity, whole brain radiotherapy

## Abstract

**Objectives:**

To develop and validate a nomogram integrating radiomic features, white matter hyperintensity (WMH) grading, and clinical factors for predicting overall survival (OS) in patients with non‐small cell lung cancer (NSCLC) and brain metastases (BMs) receiving whole‐brain radiotherapy (WBRT).

**Methods:**

One hundred and forty‐nine patients with BMs were enrolled. A radiomic score (Rad‐score) was developed based on features identified by univariate Cox regression and least absolute shrinkage and selection operator Cox modeling. WMH was graded using the Fazekas scale, into mild‐ and extensive‐burden groups. Cox proportional hazard models incorporating different combinations of these features were developed and compared. A nomogram was constructed by integrating the Rad‐score, WMH grade, and independent clinical variables, and its performance was evaluated using receiver operating characteristic (ROC) analysis, decision curve analysis (DCA), and calibration plots.

**Results:**

Twelve radiomic features were identified. Both the Rad‐score (hazard ratio (*HR*) = 1.072; *P* < 0.001) and WMH grade (*HR* = 2.420; *P* < 0.001) independently and significantly predicted OS. Significant improvements in model fit followed the inclusion of these variables (*P* < 0.01). The integrated nomogram outperformed the single‐feature models, yielding area under the curves (AUCs) of 0.820, 0.900, and 0.889 for 1‐, 2‐, and 3‐year OS, respectively, with a concordance index (C‐index) of 0.706 (95% *CI*: 0.598–0.815). Calibration plots and DCA further supported the predictive accuracy and clinical utility.

**Conclusion:**

The radiomics‐ and WMH grading‐based nomogram represents a potential prognostic tool for patients with NSCLC and BMs receiving WBRT.

## Graphical abstract

A prognostic nomogram integrating radiomic features and white matter hyperintensity (WMH) grading was developed to enable individualized survival prediction in patients with brain metastases (BMs). Integration of these imaging biomarkers into the clinical model enhanced predictive performance, indicating their incremental prognostic value for BM survival prediction.

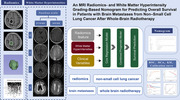

## 1 INTRODUCTION

Brain metastases (BMs) are the commonest form of brain neoplasm in adults.^1^ Lung cancer is the leading primary malignancy associated with BMs,^2^ representing approximately 50% of all patients with BM. Approximately 10%–25% of patients with stage IV non‐small cell lung cancer (NSCLC) have BMs at diagnosis.^3^ Despite ongoing advances in systemic therapies,^4^ whole‐brain radiotherapy (WBRT) has been the mainstay of BM management over the past decade, particularly in patients with multiple intracranial lesions who are ineligible for focal radiotherapy or surgical intervention.^5^, ^6^, ^7^ The overall survival (OS) of patients with BM remains limited, with the median survival less than one year.^8^ Nevertheless, survival outcomes vary considerably, from months to several years, highlighting the need for accurate prognostic assessment to facilitate individualized treatment planning.

Several prognostic scoring systems, including recursive partitioning analysis (RPA) and diagnosis‐specific graded prognostic assessment (DS‐GPA), have been established to stratify patients with BMs. However, these models were developed from cohorts treated with heterogeneous regimens, potentially introducing selection bias.^9^, ^10^, ^11^ Although repeatedly validated, they have an inherent limitation. Patients within the same prognostic category are assumed to have identical clinical features and are consequently assigned the same expected survival time. Barnholtz‐Sloan et al. revealed substantial heterogeneity in individualized survival outcomes, even within an identical prognostic category.^12^ Therefore, there is an urgent need for more individualized tools that enable accurate survival prediction in patients with BMs.

Radiomics enables the extraction of high‐throughput biologically relevant information from routine clinical imaging.^13^ Owing to the potential for early diagnosis, differential diagnosis, treatment response assessment, and prognosis prediction across various cancer types, radiomic features are regarded as promising imaging biomarkers.^14^, ^15^, ^16^, ^17^ Studies have confirmed the clinical utility of radiomics‐based survival models in oncology,^18^, ^19^, ^20^ supporting their application in personalized risk stratification of patients with BM.

White matter hyperintensities (WMH), visualized on fluid‐attenuated inversion recovery (FLAIR) imaging, are key radiological markers of cerebral small vessel disease burden.^21^, ^22^, ^23^ Owing to their frequent occurrence across a wide range of pathological conditions as well as in normal aging, WMH is an extensively investigated feature in neuroimaging. The extent of WMH has been established as a major prognostic factor for stroke recovery.^24^, ^25^, ^26^, ^27^ However, its association with prognostic outcomes in patients with intracranial neoplasms remains unclear. Given the established role of blood‐brain barrier (BBB) disruption in the development of BMs from NSCLC^28^ and the link between WMH and increased BBB permeability,^29^ it was hypothesized that a higher WMH burden would predict worse outcomes in patients with BM. If confirmed, this could contribute to an improved survival risk stratification.

This study aimed to construct and validate an integrated prognostic model incorporating magnetic resonance imaging (MRI)‐based radiomics features, WMH grading, and clinical variables to enable personalized survival prediction in patients with NSCLC and BMs undergoing WBRT.

## 2 METHODS

### 2.1 Patient recruitment

**FIGURE 1:**
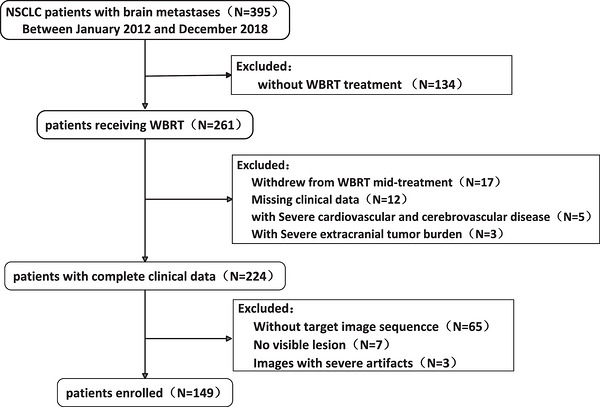
Flow diagrams showing the patient inclusion and exclusion pathways.

**FIGURE 2:**
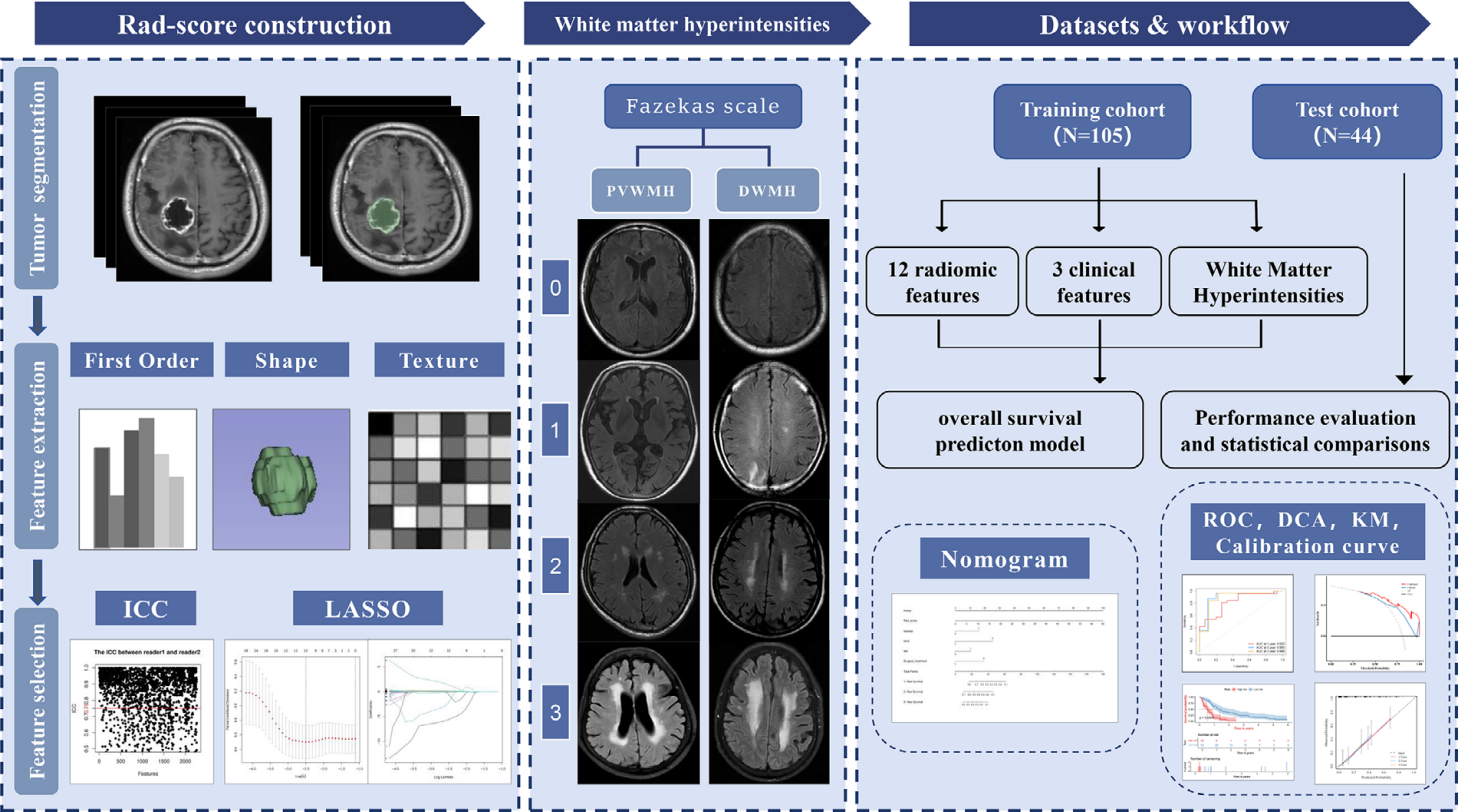
Study design and workflow.

Patients with NSCLC and BMs treated with WBRT between 2012 and 2018 were retrospectively reviewed. Inclusion criteria comprised: (1) histopathological confirmation of NSCLC through transthoracic biopsy or surgical resection; (2) pretreatment contrast‐enhanced T1‐weighted MRI acquired within 14 days, demonstrating ≥1 radiologically confirmed BM; (3) completion of prescribed WBRT regimen. The exclusion criteria were: (1) BMs from non‐pulmonary primaries; (2) severe cerebrovascular or cardiovascular comorbidities (e.g., acute ischemic stroke); (3) concurrent malignancies with significant extracranial tumor burden; (4) incomplete clinical follow‐up data; and (5) suboptimal brain MRI quality (Figure 1). The study population (n = 149) was randomly assigned to a training or test cohort in a 7:3 ratio, with model construction performed in the training cohort and validation conducted independently in the test cohort. The study workflow is shown in Figure 2. Institutional review board approval was obtained, with a waiver of informed consent (approval number: KY2025‐R195).

The main endpoint was OS, which was calculated from the initiation of WBRT to the point of either mortality or the last follow‐up. The follow‐up was completed in October 2024. Follow‐up data were extracted from institutional medical records and supplemented by structured telephone interviews.

### 2.2 Imaging acquisition and radiation treatment planning

MRI examinations were conducted on 3.0 Tesla GE (Signa HDX, General Electric, Ltd.) system configured with an 8‐channel receive‐only head coil. Patients underwent brain MRI examinations within two weeks of starting WBRT. The sequences included T1‐weighted imaging (T1WI), contrast‐enhanced T1‐weighted imaging (T1+C, a paramagnetic contrast agent injected intravenously at 2 mL/s via the antecubital vein), T2‐weighted imaging (T2WI), and T2‐weighted FLAIR. Image quality was manually assessed by an experienced neuroradiologist. For the enhanced sequences, the following parameters were applied: field of view = 230 × 200 mm; matrix = 256 × 256; repetition time = 346 ms; echo time = 5.76 ms, and flip angle = 90°. Slice thickness was set to 5 mm.

The WBRT plans were designed by radiation oncologists with extensive clinical experience. Pre‐radiotherapy planning CT scans covered C1 to 20 mm superior to the cranial vertex using 5 mm slices. WBRT was conducted using 6‐MV photons generated by an Elekta Synergy linear accelerator (Elekta Ltd., Crawley, UK). All organs at risk were delineated and adequately spared during the radiotherapy planning.

### 2.3 Image preprocessing and region of interest delineation

Several preprocessing operations were applied to the T1+C images to enhance the robustness and reproducibility of radiomics analysis. N4 bias field correction was performed to mitigate the low‐frequency intensity inhomogeneities. All images were then resampled to an isotropic resolution of 1×1×1 mm using B‐spline interpolation, whereas the corresponding region of interest (ROI) masks were resampled using nearest‐neighbor interpolation to preserve label integrity. Intensity normalization was performed using fixed‐range min–max truncation (1st–99th percentile), followed by image‐level z‐score normalization.

Tumor volumes were manually annotated as three‐dimensional ROIs on pretreatment T1+C MRI using 3D Slicer (v5.6.2). The initial segmentation by a junior radiologist was independently reviewed and refined by a senior neuroradiologist. For cases with multiple BMs, the largest lesion was selected for analysis. In cases where the tumor margins were not clearly defined, co‐registered T1WI and T2WI sequences were jointly reviewed to assist with accurate boundary delineation.

### 2.4 Radiomic feature extraction and selection

Radiomic features were extracted using the United Imaging Intelligence (uAI) Research Portal, which integrates the PyRadiomics library (https://pyradiomics.readthedocs.io/en/latest/index.html).^30^ For each ROI, 2264 radiomic features were quantified and derived from both the original images and those processed using 15 different image filters (e.g., wavelet transforms, Gaussian smoothing, and Laplacian sharpening). Key PyRadiomics settings included a fixed bin width of 25 for gray‐level discretization, extraction in 3D mode, and symmetrical gray‐level co‐occurrence matrix (GLCM) enabled. These features spanned seven radiomic classes: first‐order statistics, morphological metrics (shape and size), run length, gray‐level co‐occurrence, size zone, dependence matrices, and the adjacent gray‐difference matrix.

To assess the inter‐observer reproducibility of tumor segmentation, a subset of 30 patients was randomly selected for re‐annotation. Tumor ROIs were independently delineated by a senior neuroradiologist who was blinded to the clinical information. Interrater reliability was assessed using a two‐way random effects, single‐measure, absolute‐agreement intraclass correlation coefficient (ICC). ICCs were calculated for all extracted radiomic features, and features with ICC > 0.75 were retained for subsequent analysis.

Following z‐score normalization of the features meeting the ICC threshold, feature selection was performed in two stages in the training cohort. Univariate Cox regression analysis (P < 0.05) was used to screen variables associated with OS. Subsequently, least absolute shrinkage and selection operator (LASSO) Cox regression analysis with five‐fold cross‐validation was employed for dimensionality reduction and to identify the final predictors for model inclusion, with the optimal penalty parameter (λ) determined by the minimum criterion. The final selected features were combined to construct a radiomics score (Rad‐score), which was calculated as a weighted linear combination of the feature values and their corresponding coefficients.

### 2.5 White matter hyperintensity classification

WMH was graded on pre‐radiotherapy FLAIR images using the Fazekas scale.^31^ The Fazekas score was calculated as the sum of the periventricular (PVWMH) and deep white matter hyperintensity (DWMH) scores. PVWMH, referring to hyperintensities surrounding the lateral ventricles, was graded from 0 to 3: 0 (none), 1 (cap‐ or pencil‐shaped edge), 2 (regular hyperintense rim), and 3 (extension into the deep white matter). DWMH located in the subcortical white matter were also graded from 0 to 3: 0 (none), 1 (punctate lesions), 2 (lesions beginning to converge), and 3 (large fused areas). The total Fazekas score ranged from 0 to 6. Two experienced radiologists, blinded to the clinical information, independently reviewed the FLAIR images and assigned Fazekas scores. The inter‐rater reproducibility of the WMH grading was high, with kappa coefficients of 0.923 for PVWMH and 0.872 for DWMH.

The maximally selected log‐rank statistic was applied to determine the optimal cutoff Fazekas total score for OS in the training cohort. WMH burden was dichotomized as mild (Fazekas score ≤1) or extensive (Fazekas score ≥2).

### 2.6 Construction of the clinical model

Clinical variables included sex, age, history of hypertension or diabetes, extracranial metastases, smoking and alcohol use, receipt of chemotherapy, targeted therapy or immunotherapy, and surgical resection or irradiation of the primary tumor. Factors associated with OS were first evaluated using univariate Cox regression, followed by multivariate analysis to determine independent predictors. A clinical model was constructed on the basis of these independent predictors.

### 2.7 Survival analysis and model construction

Patients were stratified into high and low Rad‐score groups using the median score from the training cohort as the cutoff. Kaplan–Meier analysis compared the OS between these subgroups, as well as between patients with mild and extensive WMH burden. Cox proportional hazards models were used to predict OS. Five Cox models were developed: (1) radiomics only, (2) clinical variables only, (3) clinical variables plus Rad‐score, (4) clinical variables plus WMH grading, and (5) clinical variables combined with both Rad‐score and WMH grading. To evaluate and compare the predictive performance of each model at multiple time points, time‐dependent receiver operating characteristic (ROC) curves for 1‐, 2‐, and 3‐year OS were generated. Corresponding areas under the curves (AUCs) and concordance indices (C‐indices) were calculated. Calibration plots were used to compare the predicted risks with the actual survival outcomes. To evaluate the incremental prognostic value of radiomic features and WMH grading beyond clinical variables, likelihood ratio tests were performed in the training cohort by comparing nested Cox proportional hazard models. Finally, a prognostic nomogram integrating clinical variables, radiomics features, and WMH grading was constructed. A decision curve analysis (DCA) was performed to assess the clinical usefulness and net benefit of the nomogram. In addition, the prognostic performance of two established scoring systems for BMs, DS‐GPA and RPA, was evaluated by calculating their C‐indices for reference.

### 2.8 Statistical analysis

Continuous variables were compared using independent t‐tests when normally distributed or Mann–Whitney U tests; categorical variables were compared using chi‐squared tests. Predictors of OS were identified using both univariate and multivariate Cox proportional hazard regression analyses. Survival outcomes were evaluated using Kaplan–Meier analysis and log‐rank tests. Internal validation was performed using 1,000 bootstrap resamples to obtain the optimism‐corrected estimates of discrimination and calibration. Apparent and bootstrap‐corrected calibration curves were generated at 1‐, 2‐, and 3‐year time points. A two‐sided P value < 0.05 was considered statistically significant. All statistical analyses were performed using SPSS Statistics (v25.0) and R software (v4.4.1). Nomogram construction, Kaplan–Meier survival plotting, time‐dependent ROC analysis, DCA, and calculation of the concordance index (C‐index) were performed with R packages including “survival,” “survminer,” “rms,” “Hmisc,” “timeROC,” “ggplot2,” “rmda,” and “ggDCA.”

## 3 RESULTS

### 3.1 Patient characteristics

A total of 149 patients (96 males and 53 females) with NSCLC and BMs were analyzed and divided into training (n = 105) and testing (n = 44) cohorts. Mean age was 57.6 ± 10.3 years (training) and 60.3 ± 10.1 years (testing), with median OS of 10.3 months (range: 1.17–72.56) and 9.4 months (range: 1.51–86.54), respectively. The groups exhibited balanced baseline characteristics (Table 1).

**TABLE 1:** Baseline characteristics of patients in the training and test cohorts.

	ALL	Test cohort	Training cohort	
	N = 149	N = 44	N = 105	*P*‐value
Age^†^ (y)	59.5 (10.2)	57.6 (10.3)	60.3 (10.1)	0.144
**Sex**				0.955
Female	53 (35.6%)	15 (34.1%)	38 (36.2%)	
Male	96 (64.4%)	29 (65.9%)	67 (63.8%)	
**KPS**				0.817
< 70	58 (38.9%)	16 (36.4%)	42 (40.0%)	
≥ 70	91 (61.1%)	28 (63.6%)	63 (60.0%)	
**Number of brain metastases**				0.875
≥ 3	108 (72.5%)	31 (70.5%)	77 (73.3%)	
< 3	41 (27.5%)	13 (29.5%)	28 (26.7%)	
**Hypertension**				0.283
No	90 (60.4%)	30 (68.2%)	60 (57.1%)	
Yes	59 (39.6%)	14 (31.8%)	45 (42.9%)	
**Glycuresis**				0.366
No	124 (83.2%)	39 (88.6%)	85 (81.0%)	
Yes	25 (16.8%)	5 (11.4%)	20 (19.0%)	
**Smoking**				0.446
No	90 (60.4%)	24 (54.5%)	66 (62.9%)	
Yes	59 (39.6%)	20 (45.5%)	39 (37.1%)	
**Targeted therapy**				0.859
No	88 (59.1%)	25 (56.8%)	63 (60.0%)	
Yes	61 (40.9%)	19 (43.2%)	42 (40.0%)	
**Surgical treatment**				1.000
No	128 (85.9%)	38 (86.4%)	90 (85.7%)	
Yes	21 (14.1%)	6 (13.6%)	15 (14.3%)	
**Pulmonary radiotherapy**				0.464
No	118 (79.2%)	37 (84.1%)	81 (77.1%)	
Yes	31 (20.8%)	7 (15.9%)	24 (22.9%)	
**Chemotherapy**				0.916
No	33 (22.1%)	9 (20.5%)	24 (22.9%)	
Yes	116 (77.9%)	35 (79.5%)	81 (77.1%)	
**Immunological therapy**				0.632
No	144 (96.6%)	42 (95.5%)	102 (97.1%)	
Yes	5 (3.36%)	2 (4.55%)	3 (2.86%)	
**Metastasis outside the brain**				0.635
No	43 (28.9%)	11 (25.0%)	32 (30.5%)	
Yes	106 (71.1%)	33 (75.0%)	73 (69.5%)	
**Osseous metastases**				0.342
No	74 (49.7%)	25 (56.8%)	49 (46.7%)	
Yes	75 (50.3%)	19 (43.2%)	56 (53.3%)	
**Hepatic metastases**				0.125
No	140 (94.0%)	39 (88.6%)	101 (96.2%)	
Yes	9 (6.04%)	5 (11.4%)	4 (3.81%)	
**Intrapulmonary metastases**				0.749
No	137 (91.9%)	40 (90.9%)	97 (92.4%)	
Yes	12 (8.05%)	4 (9.09%)	8 (7.62%)	
**Adrenal metastases**				1.000
No	141 (94.6%)	42 (95.5%)	99 (94.3%)	
Yes	8 (5.37%)	2 (4.55%)	6 (5.71%)	
**Lymphatic metastases**				0.071
No	123 (82.6%)	32 (72.7%)	91 (86.7%)	
Yes	26 (17.4%)	12 (27.3%)	14 (13.3%)	
**Other‐site metastases**				0.749
No	137 (91.9%)	40 (90.9%)	97 (92.4%)	
Yes	12 (8.05%)	4 (9.09%)	8 (7.62%)	
**Histopathology**				0.432
Adenocarcinoma	112 (75.2%)	33 (75.0%)	79 (75.2%)	
Adenocarcinoma + squamous cell carcinoma	2 (1.34%)	1 (2.27%)	1 (0.95%)	
Other	6 (4.03%)	3 (6.82%)	3 (2.86%)	
Squamous cell carcinoma	29 (19.5%)	7 (15.9%)	22 (21.0%)	
**Location of the largest BM**				0.728
Basal ganglia region	7 (4.70%)	1 (2.27%)	6 (5.71%)	
Cerebellum	20 (13.4%)	5 (11.4%)	15 (14.3%)	
Frontal lobe	49 (32.9%)	19 (43.2%)	30 (28.6%)	
Occipital lobe	20 (13.4%)	4 (9.09%)	16 (15.2%)	
Others	7 (4.70%)	1 (2.27%)	6 (5.71%)	
Parietal lobe	25 (16.8%)	7 (15.9%)	18 (17.1%)	
Pons	5 (3.36%)	2 (4.55%)	3 (2.86%)	
**RPA**				0.879
I	21 (14.1%)	7 (15.9%)	14 (13.3%)	
II	70 (47.0%)	21 (47.7%)	49 (46.7%)	
III	58 (38.9%)	16 (36.4%)	42 (40.0%)	
**DS‐GPA**				0.547
0–1	42 (28.2%)	9 (20.5%)	33 (31.4%)	
1.5‐2	77 (51.7%)	25 (56.8%)	52 (49.5%)	
2.5‐3	26 (17.4%)	9 (20.5%)	17 (16.2%)	
3.5‐4	4 (2.68%)	1 (2.27%)	3 (2.86%)	

Abbreviations: BM, brain metastases; DS‐GPA, diagnosis‐specific graded prognostic assessment; KPS, karnofsky performance status; RPA, recursive partitioning analysis.

No variable had > 5% missingness, except for molecular subtype, which had >40% missingness and was therefore excluded from multivariable modeling.

^†^ Data are mean (standard deviation)

### 3.2 Radiomic feature extraction and rad‐score construction

**FIGURE 3:**
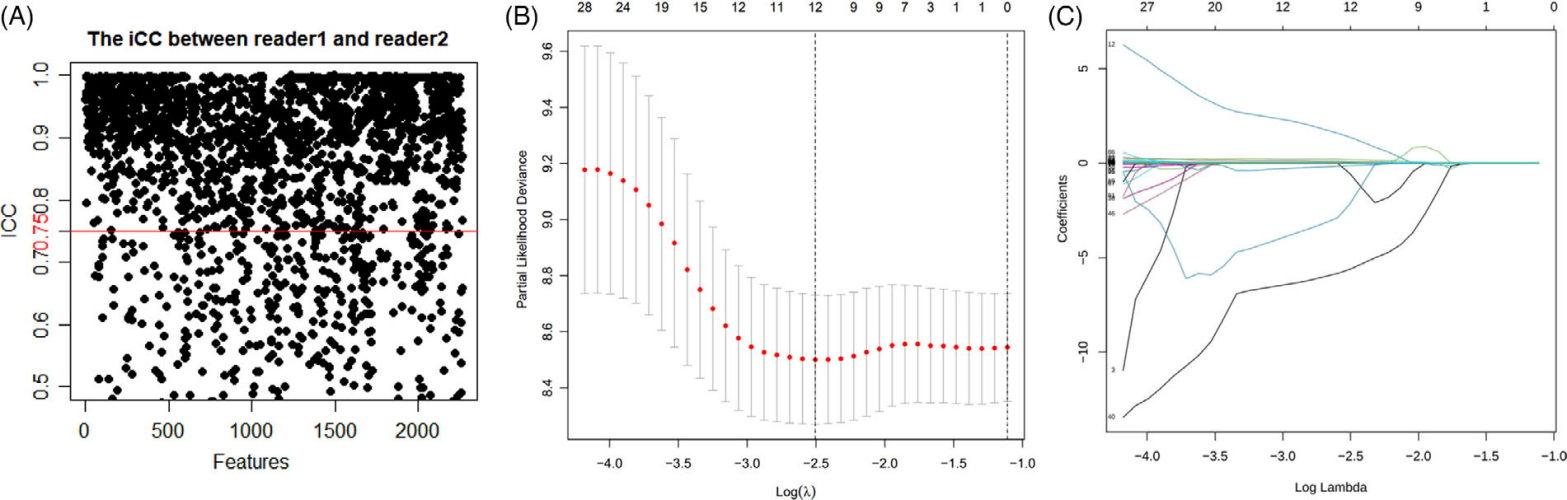
(A) ICC analysis between readers 1 and 2, (B) five‐fold cross‐validation for LASSO Cox regression, (C) LASSO coefficient profiles for radiomic features.

A total of 2264 radiomic features were extracted from each ROI. After ICC assessment, 1766 features (ICC > 0.75) were retained (Figure 3A). Univariate Cox regression identified 96 OS‐related features, which were further reduced to 12 through LASSO Cox regression (Figure 3B, C; Table S1). These 12 features were used to compute the Rad‐score using the formula provided in Supplementary Material 1.

**FIGURE 4:**
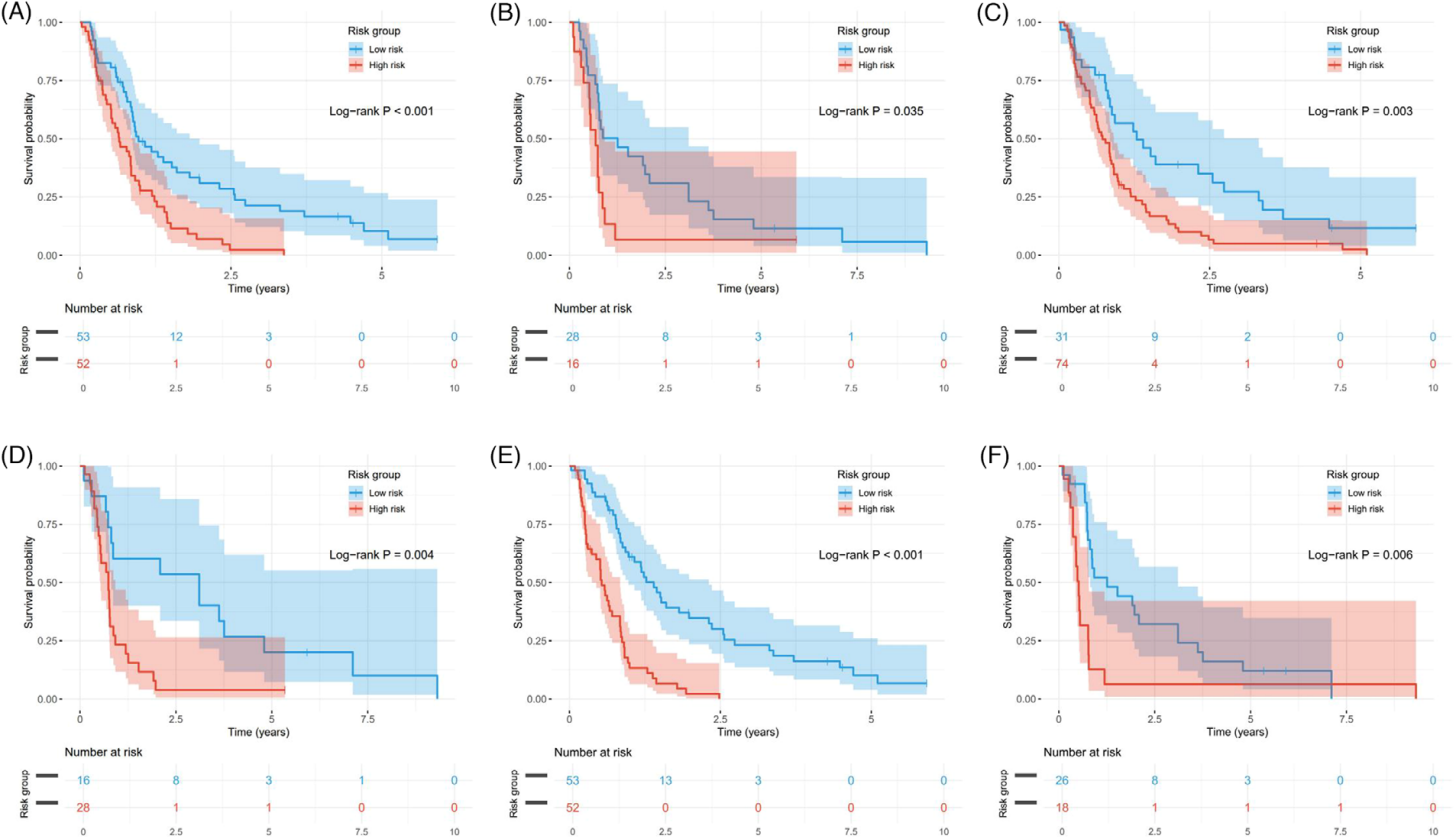
Kaplan–Meier survival curves stratified by Rad‐score in the training (A) and test (B) cohorts; Kaplan–Meier survival curves stratified by WMH grading in the training (C) and test cohorts (D); Kaplan–Meier survival curves stratified by the combined model in the training (E) and test cohorts (F).

Using the optimal Rad‐score threshold of 0.893, patients were dichotomized into high (>0.893) and low Rad‐score (≤0.893) groups. Kaplan–Meier analysis revealed a significantly worse OS in the high Rad‐score group than in the low Rad‐score group across both cohorts (Figure 4A, B).

### 3.3 White matter hyperintensity and overall survival

Among the 149 patients, 102 (68.5%) were classified as having extensive WMH (Fazekas score ≥ 2). Baseline characteristics of the mild and extensive WMH groups are summarized in Table S2. Kaplan–Meier survival analysis revealed that patients with extensive WMH had a significantly worse OS than those with mild WMH (Figure 4C, D). Both univariate and multivariate Cox regression analyses identified extensive WMH as an independent predictor of poor OS, with a hazard ratio (HR) of 2.420 (95% confidence interval (CI): 1.456–4.021, *P* < 0.001). The Cox regression analyses evaluating WMH grade and clinical risk factors in the training cohort are presented in Table 2.

**TABLE 2:** Univariate and multivariate Cox proportional hazard regression analyses of the training cohort.

Variable	Univariate analysis		Multivariate analysis (Clinical Model)		Multivariate analysis (Clinical‐WMH Model)		Multivariate analysis (Combined model)	
	HR (95% CI)	*P*‐ value	HR (95% CI)	*P*‐ value	HR (95% CI)	*P*‐ value	HR (95% CI)	*P*‐ value
KPS (≥70 vs. <70)	0.309 (0.199– 0.479)	<0.001	0.283 (0.181–0.444)	<0.001	0.259 (0.163–0.414)	<0.001	0.267 (0.169–0.428)	<0.001
Number of brain metastases (≥3 vs.<3)	1.127 (0.705– 1.801)	0.616						
Age	1.005 (0.983–1.027)	0.673						
Sex (Male vs. Female)	1.591 (1.026–2.469)	0.038	1.802 (1.142–2.842)	0.011	1.571(0.990–2.494)	0.055	1.715 (1.071–2.746)	0.025
Hypertension (Yes vs. No)	1.099 (0.723–1.669)	0.66						
Glycosuria (Yes vs. No)	0.703 (0.411–1.202)	0.198						
Smoking (Yes vs. No)	1.405 (0.914–2.162)	0.121						
Targeted therapy (Yes vs. No)	0.718 (0.466–1.106)	0.133						
Surgical treatment (Yes vs. No)	0.520 (0.280–0.966)	0.038	0.401 (0.212–0.760)	0.005	0.359 (0.190–0.679)	0.002	0.364 (0.192–0.691)	0.002
Pulmonary radiotherapy (Yes vs. No)	1.132 (0.706–1.816)	0.607						
Chemotherapy (Yes vs. No)	0.497 (0.302–0.819)	0.006	0.794 (0.462–1.364)	0.404	1.034 (0.589–1.815)	0.906	1.116 (0.629–1.980)	0.708
Immunological therapy (Yes vs. No)	0.502 (0.123–2.044)	0.336						
Osseous metastasis (Yes vs. No)	1.390 (0.901–2.145)	0.137						
Hepatic metastases (Yes vs. No)	1.375 (0.502–3,.67)	0.535						
Intrapulmonary metastasis (Yes vs. No)	1.347 (0.650–2.794)	0.423						
Adrenal metastasis (Yes vs. No)	0.790 (0.317–1.965)	0.611						
Lymphatic metastasis (Yes vs. No)	0.886 (0.482–1.630)	0.697						
Other transfers (Yes vs. No)	1.018 (0.470–2.206)	0.964						
WMH grade (extensive vs. Mild)	2.019 (1.257–3.241)	0.004	‐	‐	2.420 (1.456–4.021)	<0.001	2.30 (1.380–3.827)	0.001
Rad‐score	1.081 (1.042–1.121)	<0.001	‐	‐	‐	‐	1.072 (1.032–1.113)	< 0.001

Abbreviations: CI, confidence interval; HR, hazard ratio; KPS, karnofsky performance status; WMH, white matter hyperintensity.

The Clinical Model was constructed with significant clinical predictors from the univariate analysis (*P* < 0.05).

The Clinical‐WMH Model integrated significant clinical predictors and WMH grades.

The Combined Model integrated the significant clinical predictors, WMH grade, and Rad‐score.

### 3.4 Construction of the clinical model

In the training cohort, multivariate analysis of clinical risk factors associated with OS showed that sex (HR = 1.802, 95% CI: 1.142–2.842, *P* = 0.011), Karnofsky Performance Status (KPS) (HR = 0.283, 95% CI: 0.181–0.444, *P* < 0.001), and surgical treatment (HR = 0.401, 95% CI: 0.212–0.760, *P* = 0.005) were independent predictors of OS (Table 2). Based on these results, a clinical model was developed.

### 3.5 Model performance and comparison

**FIGURE 5:**
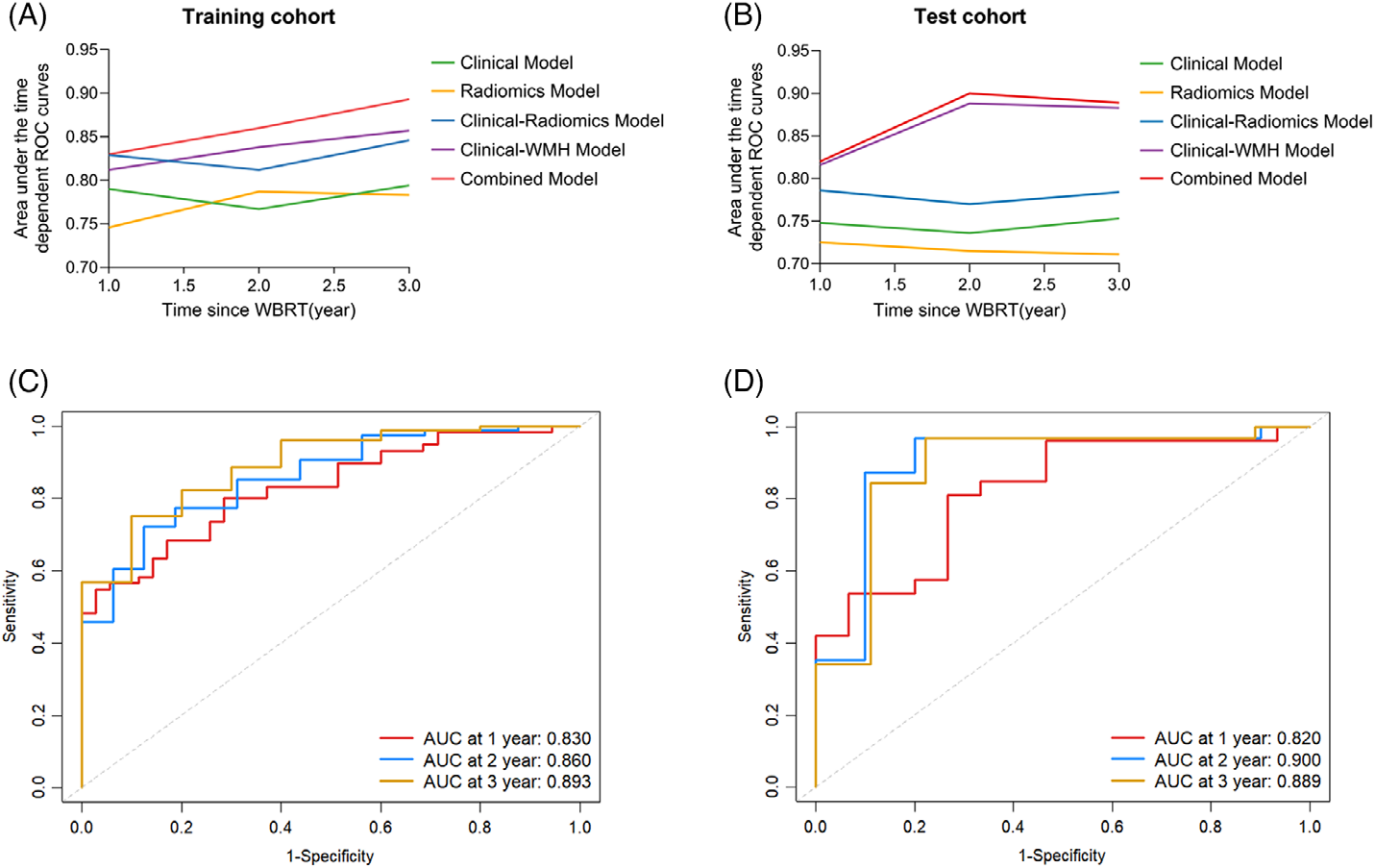
Line plots show the time‐dependent area under the curve (AUC) of different models in the training (A) and test (B) cohorts. Models were constructed using clinical variables alone (Clinical Model), radiomic features alone (Radiomics Model), combinations of clinical variables with radiomic features (Clinical‐Radiomics Model) or WMH grading (Clinical‐WMH Model), and a fully integrated model incorporating all features (Combined Model). The combined model consistently achieved the highest AUC across all time points. Time‐dependent receiver operating characteristic (ROC) curves of the combined model at 1‐, 2‐, and 3 years in the training (C) and test (D) cohorts.

Among the five Cox proportional hazards models developed, the combined model integrating the Rad‐score, clinical variables, and WMH grading showed the highest predictive performance. It achieved AUCs of 0.830, 0.860, and 0.893 at the one‐, two‐, and 3‐year time points, respectively, in the training cohort, with corresponding AUCs of 0.820, 0.900, and 0.889 in the independent test cohort. The C‐index values for the training and test cohorts were 0.738 (95% CI: 0.668–0.808) and 0.706 (95% CI: 0.598–0.815), respectively (Table 3; Figure 5A‐D; Figure S1).

**TABLE 3:** Comparison of different models using the time‐dependent area under the curve (AUC) and concordance index (C‐index)

	Training cohort				Test cohort			
Model	C‐index (95% CI)	1‐year AUC (95% CI)	2‐year AUC (95% CI)	3‐year AUC (95% CI)	C‐index (95% CI)	1‐year AUC (95% CI)	2‐year AUC (95% CI)	3‐year AUC (95% CI)
**Clinical**	0.702 (0.650–0.755)	0.790 (0.703–0.878)	0.767 (0.655–0.880)	0.794 (0.661–0.927)	0.671 (0.571–0.771)	0.748 (0.599–0.896)	0.736 (0.558–0.914)	0.753 (0.564–0.941)
**Radiomics**	0.659 (0.590–0.729)	0.746 (0.647–0.844)	0.787 (0.675–0.899)	0.783 (0.655–0.910)	0.619 (0.511–0.727)	0.725 (0.560–0.890)	0.715 (0.525–0.905)	0.711 (0.506–0.916)
**Clinical + Radiomics**	0.735 (0.680–0.791)	0.829 (0.748–0.911)	0.812 (0.703–0.922)	0.846 (0.720–0.972)	0.688 (0.589–0.787)	0.786 (0.643–0.928)	0.770 (0.591–0.949)	0.784 (0.591–0.976)
**Clinical + WMH**	0.716 (0.662–0.771)	0.812 (0.730–0.894)	0.838 (0.741–0.935)	0.857 (0.733–0.981)	0.701 (0.610–0.791)	0.816 (0.689–0.944)	0.888 (0.757–1.000)	0.883 (0.740–1.000)
**Clinical + Radiomics + WMH**	0.738 (0.668–0.808)	0.830 (0.759–0.909)	0.860 (0.781–0.946)	0.893 (0.803–0.978)	0.706 (0.598–0.815)	0.820 (0.673–0.934)	0.900 (0.750–1.000)	0.889 (0.722–1.000)

Abbreviations: AUC, area under the curve; CI, confidence interval; WMH, white matter hyperintensity; C‐index, concordance index.

### 3.6 Sensitivity and model robustness

The independent predictive value of the combined Rad‐score and WMH grade model for OS was confirmed by multivariate Cox regression (Table 2). In a sensitivity analysis additionally adjusted for the number of BMs, systemic therapy, and extracranial metastases, these covariates were not significantly associated with OS, and the hazard ratios for WMH grading and the Rad‐score remained essentially unchanged (Table S5).

Bootstrap internal validation demonstrated reasonable discrimination stability, with an optimism‐corrected C‐index of 0.728 (95% CI: 0.667–0.785). Time‐specific calibration curves are shown in Figure S2.

### 3.7 Likelihood ratio test and risk stratification

Based on the combined model's risk score, patients were stratified into high‐risk and low‐risk groups (score ≥ 0.969 and <0.969, respectively). The survival differences between these groups were significant in both cohorts (Figures 4E, F). Likelihood ratio tests indicated significant improvement in model fit with the addition of radiomic features (Δ–2LL = 10.319, *P* = 0.001) and WMH grading (Δ–2LL = 12.625, *P* < 0.001), with the combined model showing the greatest improvement (Δ–2LL = 21.297, *P* < 0.001; Table 4).

**TABLE 4:** Comparison of the Cox model fit using likelihood ratio tests in the training cohort

Model	−2LL	Δ‐2LL vs Clinical	LRT χ^2^ (df)	*P*‐value
**Clinical (Base)**	637.965	‐	‐	‐
**Clinical + Radiomics**	627.646	10.319	10.32(1)	0.001
**Clinical + WMH**	625.340	12.625	12.63(1)	<0.001
**Clinical + Radiomics + WMH**	616.668	21.297	21.30(2)	<0.001

*P*‐values were derived from likelihood ratio tests comparing each model with the base clinical model.

Abbreviations: LRT, likelihood ratio test; WMH, white matter hyperintensity; ‐2LL, ‐2 log‐likelihood.

### 3.8 Nomogram and Clinical Utility

**FIGURE 6:**
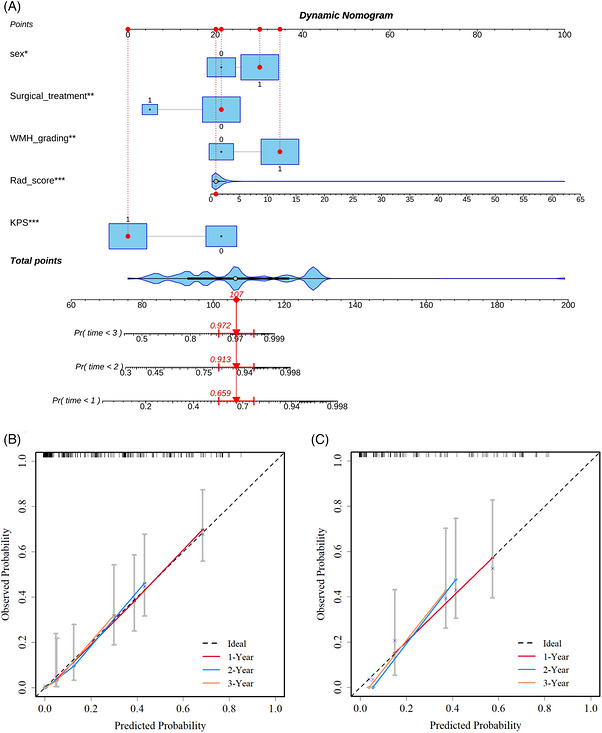
Dynamic nomogram (A) integrating the radiomic score (Rad‐score), white matter hyperintensity (WMH) grading, and clinical variables for individualized overall survival prediction. A representative patient example is displayed to illustrate the calculation of the total points and the corresponding predicted probabilities of 1‐, 2‐, and 3‐year survival. Calibration curves of the combined model in training (B) and test (C) cohorts.

**FIGURE 7:**
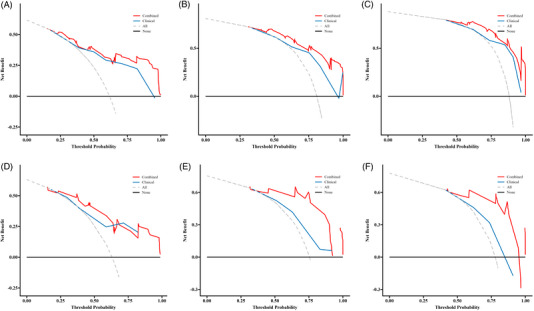
Decision curve analysis. (A–C) DCA at 1‐, 2‐, and 3 years in the training cohort and (D–F) DCA at 1‐, 2‐, and 3 years in the test cohort. Each panel compares the net benefit of the combined model (red), clinical model (blue), and two extreme strategies: treating all patients (gray dashed line) or none (black line). The combined model consistently demonstrated a higher net benefit across a wide range of threshold probabilities.

A prognostic nomogram was developed using the combined model (Figure 6A). An example of a patient is shown in the dynamic nomogram to demonstrate its clinical use in estimating individualized survival probabilities. Calibration plots for 1‐, 2‐, and 3‐year OS in both cohorts (Figures 6B‐C) demonstrated concordance between the predictions and observed outcomes. Detailed Brier scores for both cohorts, which quantitatively support model calibration, are provided in Table S3. DCA indicated that the nomogram provided a higher net benefit than the clinical model alone, across a broad range of threshold probabilities, demonstrating its potential clinical applicability (Figure 7). Finally, a comparison with established prognostic indices (DS‐GPA and RPA) showed that the nomogram achieved higher C‐indices in both cohorts, although the confidence intervals overlapped (Table S4).

## 4 DISCUSSION

This study developed and validated a prognostic nomogram incorporating MRI‐based radiomic features and WMH grading, and established clinical factors for predicting OS in patients with NSCLC and BMs treated with WBRT. The validated nomogram demonstrated acceptable predictive performance.

As survival duration increases, the likelihood of radiation‐induced neurocognitive decline also increases. Consequently, patients with a favorable prognosis are more likely to benefit from a longer‐course WBRT regimen, typically with fraction sizes less than 3 Gy.^32^ These patients are suitable candidates for treatment intensification or maintenance therapy, so there is a need for accurate survival prediction to avoid under‐treatment and over‐treatment. Rades et al. proposed a novel survival score for patients with BMs treated with WBRT,^33^ and later developed a score tailored to patients with BMs from NSCLC.^34^ However, despite the growing interest in radiomics, its application in predicting survival following WBRT for BMs remains underexplored, and current research predominantly focuses on stereotactic radiosurgery outcomes.^19^, ^35^, ^36^ Although Zhang et al. proposed a computed tomography (CT)‐based radiomic nomogram for OS prediction following WBRT,^37^ its applicability may be limited by the lower soft tissue contrast of CT. Given the status of MRI in BM evaluation,^38^, ^39^ advancing MRI radiomics for survival prediction is warranted.

Twelve radiomics features were identified for constructing the Rad‐score, and patients stratified by this score showed a significantly different OS. The radiomics–clinical model demonstrated higher discrimination than the clinical model alone, suggesting that radiomic features may provide incremental value over clinical variables, although such added value and their generalizability require confirmation in larger externally validated cohorts.

The 12 selected features were predominantly texture based. Textural features capture the spatial arrangement and heterogeneity of image intensities, offering valuable insights into tumor heterogeneity.^13^, ^40^, ^41^ The three features most strongly associated with prognosis, recursive Gaussian _glcm_Idmn, original_glcm_Idmn, and log_glcm_log‐sigma‐0‐5‐mm‐3D‐Idn, were all derived from the GLCM, which measures the spatial distribution of gray levels and reflects intratumoral texture homogeneity or heterogeneity.^42^ Given the established link between tumor heterogeneity and invasiveness,^43^ it is plausible that these GLCM features have prognostic significance. Recursive Gaussian _glcm_Idmn emerged as the most influential feature, representing large‐scale tumor homogeneity after Gaussian smoothing. This indicates that extensive regions within the tumor share similar signal intensities, which may correspond to areas of necrosis, hypoxia, or densely proliferating tumor cells. These regions are often associated with poor perfusion, aggressive behavior, and reduced sensitivity to radiotherapy, potentially explaining their negative impact on survival.

Extensive evidence has linked WMH to adverse outcomes, including stroke, cognitive decline, and mortality.^44^, ^45^, ^46^, ^47^ Debette et al. demonstrated that white matter hyperintensity volume (WMHV) is independently associated with all‐cause mortality. After adjusting for vascular risk factors, each 1 log‐unit increase in WMHV was associated with a 38% higher risk of mortality.^48^ Similarly, a low white matter grade on MRI was a significant determinant of long‐term survival in older individuals.^49^ The present study observed a similar association in the context of BMs. Although quantitative evaluation of the WMH burden can provide more detailed information, qualitative assessment enables the identification of high‐risk individuals in typical clinical settings based on the available information. The results showed that the WMH burden was independently associated with poorer OS in patients with BMs treated with WBRT. Consistently, patients with severe WMH demonstrated notably shorter survival. Notably, the WMH burden is closely related to aging and vascular comorbidities, which may confound or modify its association with survival. In the present cohort, age and major vascular comorbidities were not significant in the univariate analyses; however, this finding should be interpreted cautiously given the modest sample size, and potential interactions between WMH and vascular risk profiles cannot be entirely excluded. Although the mechanisms remain unclear, an increased WMH burden may indicate an underlying small‐vessel disease and white matter vulnerability. Such vulnerability could contribute to overall clinical fragility and potentially reduce tolerance to WBRT or other treatments, which may partly account for the survival differences observed in this cohort.^48^, ^50^ By incorporating WMH grading into prognostic modeling, this study demonstrated that the WMH burden shows exploratory prognostic relevance. Further research incorporating quantitative WMH measurements and detailed vascular profiling is required to better understand the nature and robustness of this association in patients with BMs.

Survival prediction in patients with BMs remains challenging given the influence of multiple factors such as primary tumor control and treatment, presence of extracranial metastases, number of metastases, and KPS. In this study, sex, KPS, and primary tumor resection were identified as independent predictors of OS. The KPS has long been associated with improved outcomes. Higher KPS often reflects better nutritional status and psychosocial resilience, both of which are associated with prolonged survival.^51^ David et al. analyzed 300,572 patients with stage IIIA–IV NSCLC and demonstrated that surgical resection provided significant survival benefits across all stages, with stage IV patients experiencing a markedly prolonged median OS (16.2 vs. 5.3 months).^52^ Consistent with this finding, primary tumor resection was associated with improved OS in patients with NSCLC and BMs in this study.

This study has some limitations. First, the retrospective design may introduce selection bias, and the clinical applicability of our findings require multicenter external validation. Both the training and test cohorts were relatively small compared to the high dimensionality of radiomic features, which may increase the risk of overfitting and affect the stability of the radiomics signature. Second, although all images were acquired at a single center, multiple scanners were used and no inter‐scanner harmonization was performed. This may introduce variability in the handcrafted radiomic features. Third, the current radiomics analysis was based exclusively on T1+C images and restricted to the largest lesion. Although this approach ensures methodological consistency, it may not fully capture BM heterogeneity. Fourth, molecular biomarkers of NSCLC, such as EGFR, ALK, and KRAS, were not incorporated because of substantial missing data. Given its established prognostic and therapeutic relevance, the absence of molecular information may lead to residual confounding and potentially attenuate the model's ability to fully capture biological heterogeneity. Finally, the prognostic contribution of WMH may be influenced by unmeasured vascular comorbidities or other clinical factors that were not fully captured in this retrospective dataset. Given the modest sample size and the use of qualitative WMH grading, the generalizability of its association with OS should be interpreted with caution and validated in larger prospectively collected cohorts with quantitative WMH assessment.

## 5 CONCLUSION

This study explored the prognostic relevance of WMH in patients with BMs and developed a radiomics‐based nomogram integrating the Rad‐score, WMH grading, and clinical variables. The model demonstrated a preliminary predictive value and identified distinct risk groups. These findings suggest that imaging biomarkers may provide incremental prognostic information beyond the conventional clinical factors. Multicenter prospective validations are required before clinical implementation to establish the robustness, generalizability, and clinical utility of the proposed model.

## CONFLICT OF INTEREST STATEMENT

The authors declare no financial or personal relationships that can be perceived as potential conflicts of interest related to this work.

## ETHICS STATEMENT

Ethical approval was obtained from the Institutional Review Board of the First Affiliated Hospital of Wenzhou Medical University (approval no. KY2025‐R195).

## Supporting information



Supporting Informatiom

## Data Availability

The data that support the findings of this study are available from the corresponding author upon reasonable request
