# Supplementary material for: Nomogram integrating MRI radiomics and white matter hyperintensity grading for predicting overall survival in patients with non‐small cell lung cancer and brain metastases receiving whole‐brain radiotherapy
Source: Precis Radiat Oncol. 2026 Jun 17;10(3):356–70. doi: 10.1002/pro6.70077 (PMC13398472; doi:10.1002/pro6.70077)
Supplement: Supplementary file 1 [file PRO6-10-356-s001.docx]

**Supplementary Table 1. Radiomic features and corresponding regression coefficients for Rad-score calculation.**

| Radiomic features | Regression coefficient |
| --- | --- |
| original_glcm_Idmn | β= 6.2872 |
| boxsigmaimage_gldm_DependenceNonUniformityNormalized | β= 3.7624 |
| boxsigmaimage_ngtdm_Busyness | β= 0.0004 |
| log_firstorder_log-sigma-0-5-mm-3D-10Percentile | β= -0.02121 |
| log_firstorder_log-sigma-2-0-mm-3D-Skewness | β= 0.1963 |
| log_glcm_log-sigma-0-5-mm-3D-JointEnergy | β= -0.7323 |
| log_glcm_log-sigma-0-5-mm-3D-Idn | β= -7.5199 |
| log_gldm_log-sigma-0-5-mm-3D-LargeDependenceLowGrayLevelEmphasis | β= -0.0017 |
| log_gldm_log-sigma-1-0-mm-3D-LargeDependenceLowGrayLevelEmphasis | β= -0.0023 |
| wavelet_glcm_wavelet-LHH-ClusterShade | β= 0.2635 |
| recursivegaussian_glcm_Idmn | β= -12.4501 |
| recursivegaussian_glszm_SizeZoneNonUniformity | β= 0.0659 |

Abbreviations: GLCM, Gray-Level Co-Occurrence Matrix; GLDM, Gray-Level Dependence Matrix; GLSZM, Gray-Level Size Zone Matrix; NGTDM, Neighboring Gray-Tone Difference Matrix; LoG, Laplacian of Gaussian.

GLCM (Gray-Level Co-Occurrence Matrix) Features: Calculated from the spatial relationship between pairs of pixel intensities, GLCM features capture tumor texture characteristics such as homogeneity, contrast, and correlation.

LoG (Laplacian of Gaussian) Filtered Features: Extracted from images processed with a Laplacian of Gaussian filter at a specific spatial scale, these features emphasize regions with rapid intensity changes and enhance fine-to-coarse structural details within the tumor.

**Supplementary Material 1**

Rad_score = 6.2872 * original_glcm_Idmn +3.7624 * boxsignaimage_gldm_DependenceNonUniformityNormalized +0.0004 * boxsignaimage_ngtdm_Busyness + (-0.0212) * log_firstorder_log_sigma_0_5_mm_3D_10Percentile + 0.1963 * log_firstorder_log_sigma_2_0_mm_3D_Skewness + (-0.7323) * log_glcm_log_sigma_0_5_mm_3D_JointEnergy + (-7.5199) * log_glcm_log_sigma_0_5_mm_3D_Idn + (-0.0017) * log_gldm_log_sigma_0_5_mm_3D_LargeDependenceLowGrayLevelEmphasis + (-0.0023) * log_gldm_log_sigma_1_0_mm_3D_LargeDependenceLowGrayLevelEmphasis + 0.2635 * wavelet_glcm_wavelet_LHH_ClusterShade + (-12.4501) * recursivegaussian_glcm_Idmn + 0.0659 * recursivegaussian_glszm_SizeZoneNonUniformity

**Supplementary Table 2. Baseline characteristics of patients stratified by white matter hyperintensity grading (mild vs. extensive)**

|  | **[ALL]** | **mild** | **extensive** | **p.overall** |
| --- | --- | --- | --- | --- |
|  | ***N=149*** | ***N=47*** | ***N=102*** |  |
| Age^†^ (y) | 59.5 (10.2) | 54.1 (8.06) | 62.0 (10.1) | <0.001 |
| Gender: |  |  |  | 0.512 |
| Female | 53 (35.6%) | 19 (40.4%) | 34 (33.3%) |  |
| Male | 96 (64.4%) | 28 (59.6%) | 68 (66.7%) |  |
| KPS: |  |  |  | 0.170 |
| < 70 | 58 (38.9%) | 14 (29.8%) | 44 (43.1%) |  |
| ≥ 70 | 91 (61.1%) | 33 (70.2%) | 58 (56.9%) |  |
| Number of brain metastases: |  |  |  | 0.337 |
| ≥ 3 | 108 (72.5%) | 37 (78.7%) | 71 (69.6%) |  |
| < 3 | 41 (27.5%) | 10 (21.3%) | 31 (30.4%) |  |
| Hypertension: |  |  |  | 0.262 |
| No | 90 (60.4%) | 32 (68.1%) | 58 (56.9%) |  |
| Yes | 59 (39.6%) | 15 (31.9%) | 44 (43.1%) |  |
| Glycuresis: |  |  |  | 0.260 |
| No | 124 (83.2%) | 42 (89.4%) | 82 (80.4%) |  |
| Yes | 25 (16.8%) | 5 (10.6%) | 20 (19.6%) |  |
| Smoking: |  |  |  | 0.262 |
| No | 90 (60.4%) | 32 (68.1%) | 58 (56.9%) |  |
| Yes | 59 (39.6%) | 15 (31.9%) | 44 (43.1%) |  |
| Location of the largest BM: |  |  |  | 0.101 |
| basal ganglia region | 7 (4.70%) | 3 (6.38%) | 4 (3.92%) |  |
| Cerebellum | 20 (13.4%) | 6 (12.8%) | 14 (13.7%) |  |
| Frontal lobe | 49 (32.9%) | 12 (25.5%) | 37 (36.3%) |  |
| Occipital lobe | 20 (13.4%) | 10 (21.3%) | 10 (9.80%) |  |
| Others | 7 (4.70%) | 4 (8.51%) | 3 (2.94%) |  |
| Parietal lobe | 25 (16.8%) | 7 (14.9%) | 18 (17.6%) |  |
| pons | 5 (3.36%) | 3 (6.38%) | 2 (1.96%) |  |
| Temporal lobe | 16 (10.7%) | 2 (4.26%) | 14 (13.7%) |  |

KPS, Karnofsky Performance Status; BM, brain metastases.

^†^ Data are mean & standard deviation

**Supplementary Table 3. Brier Scores of the Combined Cox Model in Training and Test Cohorts**

| **Time** | **Training Brier** | **Testing Brier** |
| --- | --- | --- |
| **1-Year** | 0.1191 | 0.1292 |
| **2-Year** | 0.1248 | 0.1396 |
| **3-Year** | 0.1121 | 0.1307 |

**Supplementary Table 4. Performance Comparison of the Nomogram, DS-GPA, and RPA Models**

| **Model** | **Training C-index (95% CI)** | **Testing C-index (95% CI)** |
| --- | --- | --- |
| **DS-GPA** | 0.605 (0.483–0.665) | 0.592 (0.514–0.695) |
| **RPA** | 0.661 (0.617–0.719) | 0.631 (0.543–0.73) |
| **Nomogram** | 0.738 (0.672–0.768) | 0.706 (0.645–0.755) |

C-index, concordance index; CI, confidence interval.

**Supplementary Table 5. Sensitivity analysis of the combined model**

| **Variable** | **Main analysis HR (95% CI), p** | **Sensitivity analysis HR (95% CI), p** |
| --- | --- | --- |
| **Rad-score** | 1.072 (1.032–1.113), <0.001 | 1.075 (1.033–1.118), <0.001 |
| **WMH grade**  **(extensive vs. Mild)** | 2.30 (1.380–3.827), 0.001 | 2.538 (1.454–4.431), 0.001 |
| **KPS**  **(≥70 vs. <70)** | 0.267 (0.169–0.428), <0.001 | 0.278 (0.169–0.457), <0.001 |
| **Gender**  **(Male vs. Female)** | 1.715 (1.071–2.746), 0.025 | 1.706 (1.056–2.754), 0.029 |
| **Surgical treatment**  **(Yes vs.No)** | 0.364 (0.192–0.691), 0.002 | 0.395 (0.197–0.793), 0.009 |
| **Number of brain metastases**  **(≥3 vs.<3)** |  | 1.158 (0.685–1.957), 0.584 |
| **Systemic therapy**  **(Yes vs.No)** |  | 1.089 (0.556–2.132), 0.805 |
| **Metastasis outside the brain (Yes vs.No)** |  | 1.242 (0.714–2.160), 0.442 |

HR, hazard ratio; CI, confidence interval; WMH, white matter hyperintensity; KPS, Karnofsky Performance Status.

**Supplementary Figure 1. ROC curves of different models in training and test cohorts.**

**(a)** Radiomics model in the training cohort.
**(b)** Radiomics model in the test cohort.
**(c)** Clinical model in the training cohort.
**(d)** Clinical model in the test cohort.
**(e)** Clinical + radiomics model in the training cohort.
**(f)** Clinical + radiomics model in the test cohort.
**(g)** Clinical + WMH grading model in the training cohort.
**(h)** Clinical + WMH grading model in the test cohort.

**Supplementary Figure 2. Bootstrap internal validation results**

Calibration curves of the nomogram for predicting 1-, 2-, and 3-year overall survival. The dashed red line represents the ideal reference line indicating perfect agreement between predicted and observed survival probabilities. The solid black line shows the apparent calibration performance, while the blue curve depicts the bootstrap-corrected calibration based on 1,000 resamples. Minor deviation from the ideal line reflects expected optimism in model estimates due to sample size constraints. The global calibration slope after bootstrap correction was 0.604.
